# Applications of step-selection functions in ecology and conservation

**DOI:** 10.1186/2051-3933-2-4

**Published:** 2014-02-07

**Authors:** Henrik Thurfjell, Simone Ciuti, Mark S Boyce

**Affiliations:** Department of Biological Sciences, University of Alberta, Edmonton, Alberta T6G 2E9 Canada; Department of Biometry and Environmental System Analysis, University of Freiburg, Freiburg, 79106 Germany

**Keywords:** Step Selection Function SSF, Resource Selection Function RSF, Resource Selection Probability Function RSPF, GPS telemetry, State-space model, Broken stick model, Habitat selection, Geographic Information System GIS, Remote sensing, Individual modelling

## Abstract

Recent progress in positioning technology facilitates the collection of massive amounts of sequential spatial data on animals. This has led to new opportunities and challenges when investigating animal movement behaviour and habitat selection. Tools like Step Selection Functions (SSFs) are relatively new powerful models for studying resource selection by animals moving through the landscape. SSFs compare environmental attributes of observed steps (the linear segment between two consecutive observations of position) with alternative random steps taken from the same starting point. SSFs have been used to study habitat selection, human-wildlife interactions, movement corridors, and dispersal behaviours in animals. SSFs also have the potential to depict resource selection at multiple spatial and temporal scales. There are several aspects of SSFs where consensus has not yet been reached such as how to analyse the data, when to consider habitat covariates along linear paths between observations rather than at their endpoints, how many random steps should be considered to measure availability, and how to account for individual variation. In this review we aim to address all these issues, as well as to highlight weak features of this modelling approach that should be developed by further research. Finally, we suggest that SSFs could be integrated with state-space models to classify behavioural states when estimating SSFs.

## Introduction

### Step selection functions, SSFs – statistical models of landscape effects on movement probability

#### Quantifying movement using SSFs

Recent progress in positioning technology has facilitated the collection of large amounts of spatial data on animals. This has led to new opportunities to investigate resource selection by animals [1, 2], but also new challenges related to the development of proper tools for the analysis of these large amounts of information [3–5]. Resource Selection Functions (RSFs) and Resource Selection Probability Functions (RSPFs) are routinely used to model habitat selection by animals using data from Very High Frequency (VHF) and Global Positioning System (GPS) locations [6–9]. A RS(P)F is defined as any statistical model deployed to estimate the relative probability of selecting a resource unit versus alternative possible resource units [6]. Satellite telemetry allows collection of accurate relocations less than 1 minute apart [10]. Spatial data collected at such high frequency open new scenarios because they contain important information about behaviour and decisions made by animals while moving through the environment [11]. Studies using such fine-scale data and dealing with animal movement and resource selection can be used to answer fundamental ecological questions related to species distributions and diversity [6, 11–13], home range formation [14], and can result in important management tools for identifying movement corridors [15], key habitats [16], and responses to disturbance [17].

A new powerful modelling approach, namely the Step-Selection Function (SSF), has been developed to estimate resource selection by animals moving through a landscape [11]. The computations required are relatively easy to carry out with tools such as GME (http://www.spatialecology.com/gme/) that works with GIS programs. The SSF is strictly related to the RSF and the RSPF. A RSF *w* of a vector of predictor covariates, **x** = *x*_1_, *x*_2_, *x*_3_, …, *x*_n,_ is any function proportional to the probability of selection of a spatial resource unit, depending on the frequency of used (f_u_) and available (f_a_) resource units. Basically, in the parametric case, a RSF is an exponential function given a sample of used and available resource units,

which corresponds to f_u_/f_a_ for any ***x***. To avoid misconception, selection is clearly based on used and available resource units, and not on used and unused ones. Compared to a RSF, a RSPF yields the actual probability that an available resource unit is selected and can be estimated using weighted distribution theory [18].

**Figure 1:**
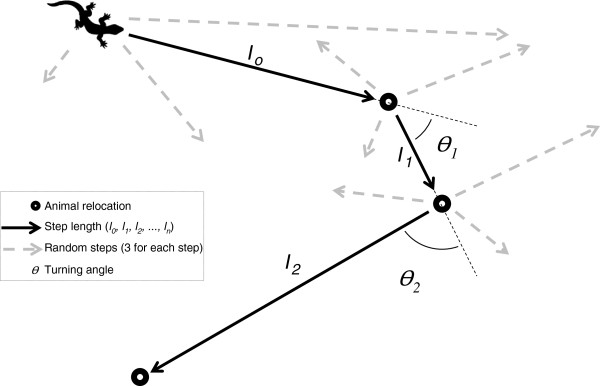
**Example of movement pathway in SSFs.** Example of how a movement pathway can be simplified into linear step lengths and turning angles occurring between successive locations in any type of animal tracked visually or using VHF or GPS devices. In this example, 3 random steps have been matched with actual steps walked by the lizard.

Including movement in selection models accommodates spatial and temporal constraints to a series of relocations, and allows the data to define the availability sample [19]. A RSF that includes movement can be estimated using an SSF [20]. Compared to RSFs, the key feature of SSFs is linking consecutive animal locations (most commonly taken at regular time intervals) that can be defined as steps [21] (Figure 1). Used steps are contrasted with a limited domain of random steps that characterize what is ‘available’ to the animal during its movement through the environment [15]. SSFs are models where each step at time *t* is paired with one or more random steps with the same starting point (i.e., matched-case or conditional approach, Figure 1) drawn at random from a distribution of step lengths and turning angles [11] (discussed in “***Calculating available steps”***). Define *μ*_*1*_*,μ*_*2,*_*…μ*_*n*_*,* to be the consecutive steps by the target animal. Let *x(u*_*i*_*) = (x*_*i1*_*, x*_*i2*_*,…,x*_*ip*_*)* denote the values of covariates (e.g., habitat characteristics) at step *μ*_*i*_. Our objective is to determine how covariates affect the selection of these steps. As for the RSF, the SSF is exponential taking the form *w*(**x**) = exp(**βx**). Previously this corresponded to f_u_/f_a_ but now for each *u*_*n*_the available units are depending on *u*_*n-1*_*,u*_*n-2*_*,K* where K is the available step drawn from a distribution of step lengths and turning angles. The main advantage of using an SSF rather than other approaches (e.g., RSFs) is that SSFs may better model selection as movement is included and constrains selection and availability [19], which enables association of parameters of movement rules with landscape features.

The aim of this paper is to review the SSF modelling approach, its applications and developments. In this first section, we clarify principal aspects of the technique. In the second section, we discuss the decisions practitioners’ face when using SSFs. In the final sections, we identify aspects of SSFs that should see further development.

## Review

### Features of step-selection functions

Here we briefly introduce main features of step-selection functions that will be fully discussed in later sections of this review.

#### Fix rate

Fix rate is the frequency of sampling, or the time between consecutive observations of location. We reviewed all studies using SSFs to model habitat selection (Table 1), and noted that fix rate has varied considerably with time intervals occurring between two successive locations ranging from 15 minutes [22] to 1 day [23]. Researchers should pay particular attention when choosing time intervals among consecutive locations because this determines the scale of possible analysis (discussed in “***Choosing the appropriate scale”***).

**Table 1:** **Review of studies that used step selection functions to model landscape effects on movement probability**

Study species	Fix-rate	# random steps	Lengths and turning angles of random steps	Modelling approach	Model validation	Ref.
Elk (*Cervus elaphus*)	5-hour	200	Drawn from 2 distributions established from observations of monitored individuals.	Conditional logistic regression	No	[11]
Cougar (*Puma concolor*)	15-min	35	Step length equal to the mean of all movement segments recorded during the same period of time. Turning angles generated at 10° increments around the starting point.	Compositional analysis	No	[22]
Roe deer (*Capreolus capreolus*)	2-hour and 6-hour	10	Drawn from 2 distributions established from observations of monitored individuals.	Conditional logistic regression	No	[46]
Elk (*Cervus elaphus*)	5-hour	20	Pairs of step-lengths and turning angles jointly sampled with replacement from empirical distributions.	Conditional logistic regression	No	[20]
Moose (*Alces alces*)	2- hour	10	Drawn from 2 distributions established from observations of monitored individuals.	Conditional logistic regression	Yes (*sensu* Boyce et al. [59])	[24]
Grizzly bear (*Ursus Arctos*)	4-hour	20	Drawn from 2 distributions established from observations of monitored individuals considering different period of the day.	Conditional logistic regression	No	[17]
Snowshoe Hares (*Lepus americanus*)	10-bound segment along hare trails left on snow	2	Drawn from 2 distributions established from observations of monitored individuals.	Conditional logistic regression	No	[28]
North Island robin (*Petroica longipes*)	1-day	10	Single dispersal step (obtained with several 1-day locations) was matched with a random walk of the same length.	Conditional logistic regression	No	[23]
Wolf (*Canis lupus*)	2-hour	25	Drawn from 2 distributions established from observations of monitored individuals at the seasonal scale.	Conditional logistic regression	No	[25]
Barred Antshrike (*Thammophilus doliatus*); Rufous-naped Wren (*Campylorhynchus rufinucha*)	15-min	20	Drawn from 2 distributions established from observations of monitored individuals.	Conditional logistic regression	No	[47]
Moose (*Alces alces*)	2-hour	2	Random turning angle (circular distribution). Random step length lower than the 99% quantile of the observed step lengths.	Conditional logistic regression	No	[64]
Moose (*Alces alces*)	1-hour	5	Drawn from 2 distributions established from observations of monitored individuals at the seasonal scale.	Conditional logistic regression	Yes (*sensu* Boyce et al. [59])	[26]
Grizzly bear (*Ursus arctos*)	1 hour	20	Drawn from 2 distributions established from observations of monitored individuals.	Conditional logistic regression (individual modelling)	No	[27]
Lynx (*Lynx canadensis*)	30-min	5	Step length and turning angle data drawn from movement paths to distinguish activity bouts from resting bouts (i.e. clusters of GPS locations).	Conditional logistic regression (individual modelling)	Yes (*sensu* Boyce et al. [59])	[16]

#### Random steps

Fortin et al. [11] defined random steps from two distributions established from observation of step lengths and turning angles of monitored individuals. Later researchers using SSFs (Table 1) limited the distributions of observed length and turning angles in an attempt to select random steps matching used steps depending on season [16, 24–26], time of day [17, 22, 27], or behaviour [16, 23, 24]. Selection of length and turning angle for random steps is likely the most critical aspect of SSFs that needs to be further developed by future research (discussed in “***Choosing the appropriate scale&Calculating available steps***”).

#### Number of random steps

Studies deploying SSFs have used various numbers of random steps matched with used steps (Table 1), ranging from 2 [28] to 200 [11] (discussed in “***Choosing the number of random steps***”).

#### Predictor covariates

Predictor covariates recorded for both used and random steps may be assessed differently depending on the research question and/or the behaviour of the species. A thorough understanding of the ecology of the species and data exploration are necessary to evaluate which attributes of the environment should be considered to explain spatial behaviours. Also, special care should be given to predictor covariates that vary both in space and time. Habitats are measured either as categorical variables such as vegetation type [11], continuous variables such as terrain ruggedness or canopy cover [11, 24], distance measures such as linear distance to roads [17, 25], or variables converted into other types of measures, e.g., resistance values [23] (discussed in “***Measuring environmental covariates, along or at endpoints of steps***”).

### User decisions

#### Choosing the appropriate scale

SSFs can be used to analyse resource selection from the second order of selection (home ranges in the landscape by monitoring dispersing individuals) [23] – to third or fourth order selection – e.g., patches within home ranges and food items within patches [29]. Both temporal and spatial scales are fundamental when modelling resource selection by animals [7], and understanding their effects is key in resource selection studies [30]. Spatial studies are strictly limited by the resolution and spatio-temporal extent of data, and it is possible to include predictor covariates measured at different scales [7]. The appropriate spatial extent in resource selection analyses depends on the research question and on the knowledge of the ecology of the target species [7, 31]. The scale needs to be fine enough to capture the ecological process or behaviour of interest, and have sufficient extent to observe the entire process or behaviour and not just a part of it. Habitat-use patterns can vary daily [32], seasonally [33], and across years [34], and the temporal extent of the analysis could be set accordingly. Boyce [7] suggested selecting the best scale by comparing alternative models, i.e., each model built using different spatial or temporal scales, by how well they predict patterns of use of the landscape. When the aim is to detect factors that limit species distributions across scales of space, multi-scale RSF modelling is strongly recommended [35]. Some processes such as predation and dispersal may consist of several processes that take place at different scales and can be depicted by RSFs estimated at multiple scales [8, 23, 36]. An example could be predator avoidance by prey that may consist of general avoidance of more risky habitats, direct avoidance of predators, or certain defence or flight strategies.

The spatial grain or resolution of spatial covariates is crucial, and spatial heterogeneity occurring at fine spatial scales can be obliterated if the resolution or grain size is too large [7, 37]. Selecting the size of sample units can be arbitrary and problematic, e.g., when one assigns to both used and available resource units a measure of road density estimated in areas of 1 ha, 1 km^2^, and 10 km^2^. Also in these cases, alternative models might be built with covariates recorded at different spatial scales and then evaluated using metrics such as the Akaike Information Criterion (AIC) [7]. If spatial data are too fine scaled, selection that takes place on a larger scale might be difficult to detect. Also, there is a temporal aspect to some spatial covariates (e.g., vegetation productivity in a given pixel of the landscape), and both sampling and model building must accommodate spatial covariates varying in time [38].

**Figure 2:**
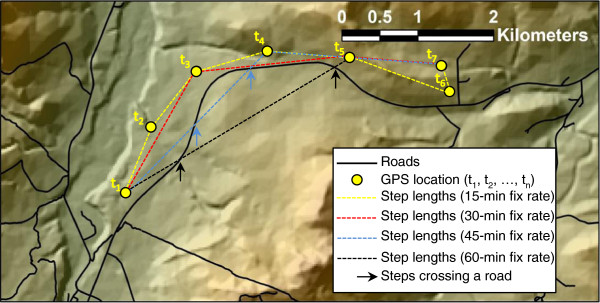
**Fix rate can affect habitat patterns revealed by SSFs.** A hypothetical terrestrial mammal is tracked with a GPS device with a 15-min fix rate. With this sampling regime, steps never cross linear features such roads, and the SSF would likely depict avoidance of roads by the animal. The same applies with a 30-min fix rate. However, 45-min or 60-min fix rates result in steps that cross roads. In this case, the fix rate is expected to affect parameter estimations, and, specifically, to influence the final pattern of selection for roads recorded for the target species (e.g. selection for roads). The opposite scenario could occur with very high fix rates, say 2-min: if this is the case, steps would be so short that either steps walked by the animal and random steps do not cross the road, and no selection or avoidance for roads would be found. Assessing the proper fix rate depending on the ecology of the species and the biological question seems to be fundamental to understand animal movement patterns properly.

Fix rates (i.e., time between successive locations) decide the temporal and spatial scale in SSFs. Nams [39] suggested that there is a natural scale of fix rates, where the time between consecutive steps represents a new choice or activity, and different behaviours act on different scales [40, 41]. This results in different observation scales (e.g., fix rates) needed to study various behavioural processes and the optimal fix rate(s) depends on the research question(s) [7]. Even behaviours that seem to act on large scales, such as dispersal, might be a series of choices made at finer scales [23], e.g., step after step. On the other hand, ecological processes that are evident at the finer scale could be less clear when looking at a larger scale [7]. Because fix rate decides the order and strength of habitat selection, the optimal fix rate should be evaluated carefully before conducting telemetry studies. At high fix rates, where the average step length is shorter than five times the locational error, both step length and turning angle could be overestimated [42]. A fix rate that is higher than necessary for the scale of selection also can lead to misleading results because avoided habitats might not be included in the random samples. In the example illustrated in Figure 2, the use of 15-min or 30-min fix rates could allow the researcher to depict avoidance of roads by a hypothetical species patrolling the landscape without crossing roads. In contrast, 45-min or 60-min fix rates could produce steps artificially crossing roads and likely affecting SSF parameter estimation (Figure 2). On the other hand, if we imagine a 1-min fix rate, steps would be so short that both used and available random steps would never cross a road, with no chance of depicting avoidance or selection of roads by this species. To avoid analysing resource selection patterns on the wrong scale, we recommend performing pilot studies with as high fix rates as possible considering locational error [42] and then assessing the data and testing models with successively lower fix rates, either using Information criterion or wavelet analysis [43]. If the main goal of the research is to understand response to roads by the target species (Figure 2), then the researcher could run several preliminary SSFs using 15 min fixes and artificially reducing fix rates (e.g., 30-min, 45-min, 60-min, …, n-min), and then obtaining parameter estimates for each simulation. Estimated parameters for the selection of roads would likely follow a pattern with a turning point (e.g. between 30-min and 45-min fix rates) at which avoidance of roads would change, and this could be used to determine the lowest fix rate for that specific research question. Clearly this technique needs to be developed by further research. Modern GPS units are more flexible, with fix-rates easily controlled remotely, and this opens new possibilities to save battery life and still have data that are adequate to meet the needs of the research question.

#### Calculating available steps

Because SSFs compare use versus availability, the methods for generating available steps are crucial. Random steps can be generated either from empirical or parametric distributions [20], or possibly simulated within the framework of movement models (see “***New directions for developing SSFs***” for further discussion).

For steps drawn from empirical distributions, the most common way has been to proceed using the method of Fortin *et al.*[11] to avoid issues of circularity, i.e., for each monitored individual draw random step-lengths and turning angles independently from two empirical distributions built with data collected from other monitored individuals from the same population. By doing this, we make the assumption that all sampled animals have similar behaviour, and also that animals make their movement choices depending on resource availability within the reach of one step length. However step length and turning angle cannot always be considered independent [44]. The correlation between the step length and turning angle depends on the fix-rate and the behaviour of the species, as we show in Table 2. A high fix rate appears to increase the correlation between step length and turning angle because one step will represent a part of behaviour such as foraging or moving between patches instead of the only representation of that behaviour during its duration. For elk (*Cervus elaphus*), the correlation between step length and turning angle is relatively weak even at a 2-hour fix rate during the migration period when correlation should be at its highest due to more directional movement (Table 2), probably because elk have relatively short duration of movements relative to fix rate. For cougars (*Puma concolor*), a species that makes long directional movements and then may have clustered positions when eating prey, the correlation between step length and turning angle is high (Table 2) and decreases slightly when fix rate increases from 15 min to 3 hr.

**Table 2:** **Relationship between step lengths and turning angles along movement path recorded for cougars and elk**

Species	Fix-rate	Mean r^2^	Max r^2^	N	Method	Sign of the relationship	Source
Cougar^1^	3-hour	0.11	0.16	4	Linear regression^4^	-	Banfield et al., unpublished data
Cougar^1^	15-min	0.17	0.22	7	Linear regression^4^	-	Banfield et al., unpublished data
Elk^2^	5-hour	NA	< 0.03	11	Correlation	NA	[11]
Elk^3^	2-hour	0.02	0.07	73	Linear regression^4^	-	Thurfjell et al., unpublished data

^1^from January to December – SW Alberta, Canada.

^2^winter – Yellowstone National Park, Wyoming, USA.

^3^during spring migration – SW Alberta, Canada.

^4^dependent variable: log-transformed step length; independent covariate: absolute turning angle.

Some researchers have instead chosen to sample available locations based on parametric distributions [20]. This assumes that animals make their movement choices based on the distribution used. A uniform circular distribution for the angle would for example assume animals have knowledge of everything within the distance of a step in all directions. Different choices on how to select step length and turning angle will affect the analysis or quantification of selection. Forester et al. [20] showed that less realistic sampling is more biased and that inclusion of step length as a predictor covariate reduces this bias, therefore recommending that step length is always included. We believe that striving for the strongest selection coefficients may not always be the answer to biologically relevant questions. The results that come out of realistic distributions, i.e., paired turning angles and step lengths or a more realistic parametric distribution might reflect the choices made by the animals better [20], even if the selection coefficients are weaker. For future studies we therefore recommend that the correlation between step length and turning angle be estimated before fitting the SSF. If the correlation is high, as might be the case with high fix rate or predators patrolling the environment (Table 2), step length and turning angle should be drawn in pairs [20].

#### Choosing the number of random steps

A small number of available samples can influence coefficient estimates potentially causing misinterpretations of habitat selection patterns [45]. However, this is not a concern in resource selection analyses using conditional regression approaches, such as for SSFs, for which the number of available samples (i.e., random steps) can be low with no effect on parameter estimation. Fortin et al. [11] used 200 random steps because their research question was to detect selection for rare habitats; however, such a large number of available random steps is generally not needed to estimate a SSF [45]. If sample size is relatively large, a large number of random steps can make the size of the database excessive, resulting in computational limitations imposed by computer power and processing time. Because most datasets generated by GPS radiotelemetry have a large number of locations per animal, often thousands, we suggest that for most cases a low number or even one random step per used step could be sufficient [45].

#### Measuring environmental covariates, along or at endpoints of steps

Steps can be characterised by the lines between locations, the average of continuous variables along the step [11], extreme values of continuous variables along the step [11], the proportion of habitats along the step [11], or with habitats measured at intervals along the step [46]. Another way to characterise steps is by the environmental features of the endpoint of the step [11, 17, 47]. Buffers also can be applied to steps or endpoints and covariates measured within those buffers [22, 46].

**Figure 3:**
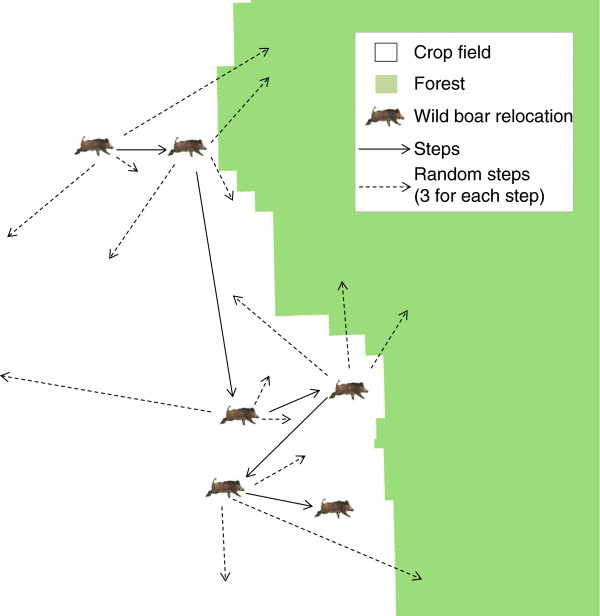
**Habitat measurements along habitat edges in SSFs.** Hypothetical relocations of a wild boar foraging along the edge of a crop field (*sensu*[48]) – steps and 3 pair-matched random steps have been reported in the figure. If habitat is measured only as field or forest, then forest habitat will most likely be avoided by wild boar in an SSF analysis. However, for safety reasons (i.e., lower probability of being detected by hunters) the wild boar is foraging close to the forest edge rather than in the middle of a crop field. The mistake by the researcher might be neglecting the perception of the habitat by the animal, assuming that all areas of the crop field are of equal quality for the wild boar. Adding ”distance to forest edge” as an attribute of the quality of crop fields is one way to catch the selection by wild boar of areas of the crop field located along the forest edge.

The difference between measuring covariates along steps and at endpoints will be greatest when animals in some way react to linear spatial covariates. The endpoints of the steps are known to be an actual relocation compared to the covariates measured along the linear steps, burdened by the assumption that the animal moved in a straight line between the 2 points. When the landscape contains linear features that might affect animal behaviour, such as roads, corridors, edges or streams, special consideration needs to be taken to analyse those correctly. For example if we consider a wild boar (*Sus scrofa*) foraging at the edges of a crop field while staying in the relative safety of the vicinity of the forest (Figure 3, *sensu*[48]): assuming that an appropriate fix rate has been chosen, an SSF will show a stronger avoidance of forest than it would for a species using the central sector of the field far from the forest edge. This is because the selection depends on the likelihood that a random step ends within the forest (Figure 3). When we have to deal with such behavioural patterns, categorical habitat measures such as “field” or “forest” are not sufficient. Instead, distance to forest edge or similar continuous covariates might be considered to better characterise wild boar foraging behaviour and to document its attraction for open areas close to a forest edge (Figure 3).

**Figure 4:**
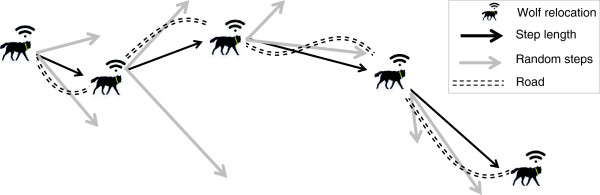
**Dealing with linear features in SSFs.** Hypothetical GPS relocations of a wolf walking along a gravel road (*sensu*[49]). SSFs could underestimate selection for roads by the wolf if landscape features are measured along the lines between steps. Habitat measured at the end point of the step (wolf relocated on the road) could allow for better depiction of selection for roads by the wolf, because random steps will be less likely to end on roads. Note that many roads and other linear features are mapped as vectors without a surface, meaning that it is impossible that a wolf location will be exactly located on the road in a GIS framework. The use of buffer areas around the endpoint or, alternatively, the distance of the step endpoint to the linear feature are good ways to capture selection of linear features by animals.

In cases where linear elements are preferred and narrower than the measured step length, as for wolves (*Canis lupus*) using gravel roads as movement routes [49], using the lines between steps rather than the end points of the steps might underestimate selection for roads. Only a small portion of the line will be on the road because the lines are straight and the road is not (Figure 4), even if the wolf is actually on the road the entire time. If linear elements are instead avoided, only steps or buffers along steps, and not the end points will be able to catch the crossings of such objects [16]. Note that many linear elements are line features in a GIS environment, containing no surface, therefore it is impossible for point locations (e.g., the end of a step) to end up exactly on them.

In most studies large-scale maps, remote sensing or satellite imagery with low resolution are used as a source of environmental variables for obvious practical reasons and limited budgets, especially when target species are relocated across large regions. To answer more fine-scaled questions however, these data layers may not have the necessary resolution [48]. Modern real-time GPS radiotracking allows frequent downloads of data which in turn can be analysed in SSFs throughout the study. This enables researchers to collect field data from real and random steps by visiting them and measuring, e.g., biomass, vegetation species composition, etc. (close in time to avoid seasonal changes in environmental covariates). Care must be taken not to disturb radiocollared individuals during data collection because this might obviously skew the results.

#### Statistical tools for SSFs

Similarly to a Resource Selection Function [6], a Step Selection Function SSF usually takes the exponential form

where *β*_*1*_ to *β*_*p*_ are coefficients estimated by conditional logistic regression for associated covariates *x*_1_ to *x*_*p*_, respectively [11]. Steps with a higher SSF score *w(****x****)* have a higher likelihood of being chosen by the tracked animal. For two normal distributions (i.e., distributions of available and used resources), the exponential model provides the correct form of the RSF, but for other distributions, logistic or probit models might best fit the data (see [9]).

Almost all studies to date have built SSFs using conditional logistic regression (Table 1), with only a few exceptions (e.g., compositional analysis [22]). Duchesne et al. [50] showed the importance of using mixed conditional logistic regression in matched use-available habitat selection designs. Specifically, Duchesne et al. [50] showed how mixed conditional logistic regression could be used in the presence of among-individual heterogeneity in selection, and when the assumption of independence from irrelevant alternatives (IIA, [51]) is violated. Despite their suggestions, since their publication no studies to date have used mixed conditional logistic regression to model SSF - but see Gillies et al. [47] and Forester et al. [20] who took into account among-individual variation. This could be related to the limited availability of software for calculating mixed conditional regression: this can be done in Matlab [50] or in R [52] by i) doing a re-parameterization of a *lmer* (linear mixed model *lmer*, *lme4* package) to a conditional model, i.e., a model with no intercept where the variables are expressed as the difference between the paired used and available, ii) using the *coxme* function (*coxme* package) by setting time equal to 1 for all data points, or using the *mclogit* package.

An alternative to mixed-modelling is individual modelling, as done by Squires et al. [16] and Northrup et al. [27] for SSFs. Individual differences in behaviour, including habitat choices, have become a key target of research with important ramifications for ecology and evolution [53]. Resource selection can have strong inter-individual variability within a population in response to several factors [54]. With abundant relocations, GPS units generate enough data to fit individual models.

A method for fitting individual resource-selection models, and to obtain models for inference at the population level, is the two-stage modelling approach [4]. The first stage involves fitting, ranking [55] and averaging *a priori* models [4, 56, 57] separately for individual animals. The second stage is to average regression coefficients across individuals to estimate population-level selection [57]. This can be done either manually or using routines provided by the *TwoStepClogit* package in R. Fieberg et al. [4] recommend the two-stage approach as a practical method to account for correlation within individuals in habitat-selection studies.

**Figure 5:**
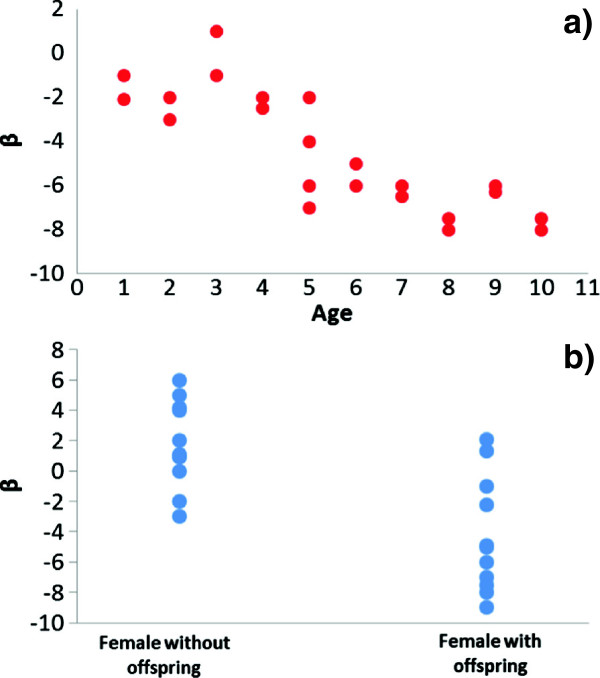
**Individual modelling in SSFs.** SSF estimates computed at the individual level can be further analysed with common statistical packages to make inferences about the effects of additional covariates on habitat selection. In example **a)**, age of monitored animals are plotted on the x-axis, while individual selection coefficients β estimated with SSFs (say selection for roads) are plotted on the y-axis. In this case, there is a clear increase in the avoidance of roads in older individuals, and this pattern can be analysed with a linear regression, a generalized linear model, or a generalized additive model. In example **b)**, selection coefficients estimated with SSFs (say selection for open areas) are plotted for females with or without offspring. The effect of offspring on selection for open areas by mothers can be tested with an independent sample t-test, for instance, or using generalized linear models if other covariates are available (say the age of the female).

The first stage allows for subject-specific inferences and variance decomposition between and within groups, and, more importantly, can accommodate variable habitat selection responses among individuals [4]. Coefficients estimated for each individual can be analysed to portray personality traits [53], or to test specific hypotheses on the behavioural ecology of a target species, e.g., functional responses in habitat selection [8]. For instance, individual estimates of beta coefficients can be processed using conventional statistical packages (e.g., linear and non-linear regression, generalized linear models GLMs, and generalized additive models GAMs) to test the effect of continuous covariates such as body weight or age on habitat selection (Figure 5a). Other statistical tools (e.g., independent sample t-tests) also can be used to test for variation in beta values estimated in animals characterized by different reproductive status (e.g. female with offspring *vs.* females without offspring, Figure 5b), movement strategy (e.g., migratory *vs.* non-migratory), or future survival (e.g. depredated individuals *vs.* survivors).

With increasing fix rate, positional data of animals also becomes increasingly autocorrelated in time [58]. This will not affect the beta estimates but will result in underestimated variance for these estimates [7]. Fortin et al. [11] dealt with temporal autocorrelation by calculating and correcting the confidence intervals based on rarefied data where locations are no longer correlated. Another way to account for temporal autocorrelation is to include an autocorrelative structure [26] or the temporal variables as predictor covariates. Often the autocorrelated nature of the landscape explains the autocorrelation in the data and one can evaluate this by fitting the model and examining the residuals for autocorrelation. In many instances we have found that the residuals are not autocorrelated.

Before applying such models in management and conservation plans [59], evaluation of model performance is a necessary but commonly neglected procedure in resource-selection studies, and this applies to SSF studies as well (Table 1). Although a number of methods are available for presence-absence data (e.g., [60, 61]), these evaluation approaches are not appropriate for presence-available designs because presence sites are derived from the distribution of available sites [59, 62, 63]. A k-fold cross-validation method should be appropriate for SSF designs and could be used to verify the accuracy of predictions such as previously done for RSFs [59, 63]. We encourage further research to develop new evaluation methods to ensure that predictions from SSFs models are robust before using them to plan conservation actions.

### Applications of SSFs in ecology and conservation

Predictions of SSF portrayed in the GIS environment are probably one of the most promising tools in ecology, management and conservation. SSFs are a powerful technique for identifying the habitats that animals choose to move through, expanding our knowledge of animal decision-making at finer spatial and temporal scales. This approach has the potential to be widely used to understand animal behaviour within human-dominated landscapes, e.g. to assess the effect of human disturbance on wildlife [64, 65], to predict movement corridors in human-dominated landscapes [16, 17, 23], and to plan management and conservation strategies accordingly. SSFs are particularly useful for understanding the effects of human-related features such as roads and associated vehicle traffic [11, 17, 27]), the use by wildlife of man-made linear features [25], and relationships between temporal patterns in human activity and consequent disruption of animal behavioural patterns [64, 65]. SSFs combined with cost-distance modelling can assess functional landscape connectivity [23] and dispersal behaviour [17] by considering entire dispersal events and a random walk of similar properties as the alternative step(s) [23]. Squires et al. [16] used RSFs to find potential animal home ranges, and then SSFs and least-cost-path models to define movement corridors between the potential home ranges by mapping SSFs. The map identified dispersal corridors for Canada lynx (*Lynx canadensis*) made by plotting the SSF-values, rescaled to relative probability of use between 0 and 1, excluding the 5% highest and lowest values to remove outliers [16]. This is a promising development of the technique, with great potential for management and conservation planning. Parameters of SSFs could be artificially modified to create scenarios within GIS framework for conservation plans, e.g., by artificially increasing road density or deforestation and to verify how habitat selection predicted by SSFs changes.

### New directions for developing SSFs

There are several other potential ways in which steps could be calculated for assessing functional landscape connectivity. For example spatial graph-theoretic approaches such as Brownian bridges or circuit theory might be used to define steps instead of the straight lines between observations, and could be used for generating random steps as well [43]. Broken-stick models [66], transition equations [44], and state-space models SSMs [67] are approaches taking into account that different behaviours shape movement parameters. These approaches can be integrated with SSF designs to develop new resource-selection models within the same framework. Specifically, they could be excellent methods for defining the length and turning angle of random steps depending on the state or behaviour of the animal. This is likely the most critical point of SSF models, because it is clear that selection patterns depend on how we choose available resources.

In broken-stick models, each step can be assigned to a behaviour such as intra-patch foraging, inter-patch movement or migration [66]. With transition equations, the possibility of an animal changing behavioural state from one to another is calculated [44]. In state-space models, the previous step of the animal determines the likelihood of the next step, based on its location and on the properties of previous steps, usually via a Markov chain [67]. State-space models also have the advantage of accounting for the observational/locational error in the observation model [67]. SSFs can be improved by combining these models in several ways. A broken-stick model can objectively distinguish different types of behaviours [66], and the distribution of random step lengths and turning angles can be drawn within those behaviours [16]. This could account for the correlation between step length and turning angle because they would be drawn from populations of observations within each behaviour, and one SSF could be produced per behaviour (see [16] for an example where a single behaviour was tested).

Another approach would be to estimate the random steps within the framework of the state-space model [67] by estimating the random steps based on previous steps to determine the behaviour distribution (D_n_) from which the random steps should be drawn. If a vector of distributions (**D**) represent one behaviour each, and a number of transition equations (**T**) represents the chance of an animal going from one behavioural state to another given a number, *n*, of previous locations (*u*_*t*-1_…*u*_*t-n*_). The function of available units could look like *f*(*a*_*u*_(*t*-1, *t-n*)_,_**D**,**T**). This would associate each step with random steps accounting for the possibilities that the animal continues with its current behaviour or changes to a new behaviour [44]. In this way each position is associated with the choices the animal is faced with. An example would be a lion (*Panthera leo*) that has just eaten, as shown by the properties of the steps. The probability for the following steps to be searching for prey is low and for resting and digesting is high. As the time from the feeding increase, the probability of steps belonging to a search behaviour increases because the lion will get hungrier.

## Conclusions

SSFs have a distinct advantage over regular RSFs because they include the serial nature of animal relocations and can associate parameters of movement rules with landscape features, and they can model the choices actually presented to the animal as it moves through the landscape [15]. However, as strong as the tool might be, there are several pitfalls that must be avoided in order to accurately capture behaviours and ecological processes. The properties and scale (fix rate) of steps (lines or endpoints), and the habitat measurements that are taken must be able to capture the relevant behavioural processes, and we recommend that analyses are carried out after thorough data exploration and with good knowledge of the behaviour and ecology of the target species.

So far few studies have taken into account the differences among individual animals. Mixed conditional models are one way to deal with this source of variability, especially if the sample size is moderate. However, if the data are sufficient to allow it, we believe individual modelling has more advantages, is simpler to carry out in conventional software, and has the potential to capture ecological processes that are considered random variation in conditional mixed-effects models.

A fix rate that has both the resolution and temporal extent to capture the studied behaviours is necessary, and we strongly recommend that researchers start by considering which scale they are interested in and at which scale they will access the covariate data. Then they can try with a fix-rate that is slightly high and do several preliminary analyses with rarefied data. Then they could re-set the fix rate to balance the trade off between a high fix rate and a long battery life of the GPS unit. As fix rate increases, the probability of autocorrelation between step length and turning angle will increase, and the influence of positional errors increase. This needs to be tested before further analysis is carried out; we recommend either to include this correlation in the process of selecting random steps or to assign behaviours to each step as per the broken-stick model and estimate one SSF per behaviour. In the future we believe that these processes could be integrated by using SSMs in the process of selecting random steps and thus to estimate SSFs where selection of a movement path depends on the positional locations themselves and the state of the animal.

## Abbreviations

SSF: Step selection function
RSF: Resource selection function
RSPF: Resource selection probability function
VHF: Very high frequency
GPS: Global positioning system
GIS: Geographic information system
SSM: State space model.
